# The Functional Response of B Cells to Antigenic Stimulation: A Preliminary Report of Latent Tuberculosis

**DOI:** 10.1371/journal.pone.0152710

**Published:** 2016-04-06

**Authors:** Willem J. du Plessis, Léanie Kleynhans, Nelita du Plessis, Kim Stanley, Stephanus T. Malherbe, Elizna Maasdorp, Katharina Ronacher, Novel N. Chegou, Gerhard Walzl, Andre G. Loxton

**Affiliations:** SA MRC Centre for TB Research, DST/NRF Centre of Excellence for Biomedical Tuberculosis Research, Division of Molecular Biology and Human Genetics, Faculty of Medicine and Health Sciences, Stellenbosch University, Cape Town, South Africa; University of Cape Town, SOUTH AFRICA

## Abstract

*Mycobacterium tuberculosis* (*M*.*tb*) remains a successful pathogen, causing tuberculosis disease numbers to constantly increase. Although great progress has been made in delineating the disease, the host-pathogen interaction is incompletely described. B cells have shown to function as both effectors and regulators of immunity *via* non-humoral methods in both innate and adaptive immune settings. Here we assessed specific B cell functional interaction following stimulation with a broad range of antigens within the LTBI milieu. Our results indicate that B cells readily produce pro- and anti-inflammatory cytokines (including IL-1β, IL-10, IL-17, IL-21 and TNF-α) in response to stimulation. TLR4 and TLR9 based stimulations achieved the greatest secreted cytokine-production response and BCG stimulation displayed a clear preference for inducing IL-1β production. We also show that the cytokines produced by B cells are implicated strongly in cell-mediated communication and that plasma (memory) B cells (CD19^+^CD27^+^CD138^+^) is the subset with the greatest contribution to cytokine production. Collectively our data provides insight into B cell responses, where they are implicated in and quantifies responses from specific B cell phenotypes. These findings warrant further functional B cell research with a focus on specific B cell phenotypes under conditions of active TB disease to further our knowledge about the contribution of various cell subsets which could have implications for future vaccine development or refined B cell orientated treatment in the health setting.

## Introduction

*Mycobacterium tuberculosis* (*M*.*tb*) is the causative agent of tuberculosis (TB) disease and is responsible for great annual morbidity and mortality. Furthermore, the World Health Organization (WHO) reported that there were 9.6 million newly reported cases of TB coupled with 1.5 million reported deaths in 2014 [1]. Although great progress has been made in delineating the disease coupled with the use of an effective chemotherapeutic regimen, TB remains endemic with the growing threat of MDR-TB (multidrug-resistant TB) and XDR-TB (extensively drug-resistant TB) [2].

A balance between regulatory and effector immune responses is in most cases sufficient to contain an *M*.*tb* infection to what is commonly known as latent tuberculosis infection (LTBI), as it’s estimated that only 1 out of every 10 people infected with the pathogen will progress to active disease [3, 4]. The lack of vaccines capable of preventing active TB places a lot of pressure on controlling the epidemic [5, 6]. Numerous studies were performed in attempts to identify biomarkers not only to assist in the accurate and timely diagnosis of TB, but also to fuel the development of TB vaccines and drugs [7, 8].

The prevalence of LTBI remains high, especially in household contacts of TB patients in highly endemic settings, proving that the molecular mechanisms that constitutes and maintains the abovementioned immunologic balance between protection and/or disease progression is not well understood. Working towards understanding these mechanisms is challenging considering the overwhelming complexity observed between the biologic interaction of the host, microbe and environment [9, 10]. As suggested by Barry *et al*, LTBI and active TB disease represents a spectrum of disease states rather than being mutually exclusive [11]. This is supported by work done by Lin *et al*. in a cynomolgus macaque model [12]. Although the factors that mediate this spectrum are not well understood, it is quite possible that the host is protected from the progression of LTBI to active TB by the enrichment of potent anti-*M*.*tb* specific effector cells [13].

Human tuberculosis is primarily controlled by the activation and infiltration of CD4^+^ Th1 cells and CD8^+^ cytotoxic lymphocytes [14]. Although their involvement is still considered controversial [15], B cells have proven to contribute to TB immunity in various ways.

Some of these B cell functions include presenting antigens to naïve T cells in the *M*.*tb* infected lung [16, 17], antibody production [18, 19] and cytokine production [20]. A unique effector subset of B cells (described as innate response activator (IRA)-B cells) were identified as the primary producers of granulocyte macrophage colony-stimulating factor (GM-CSF) during experimental sepsis [21]. More recently, another innate effector B cell subset was identified and implicated in the promotion of favourable Th1 responses by interferon (IFN)-γ production [22]. Additionally B cells are unable to control infection with intracellular bacteria (including *M*.*tb*) [23, 24], yet they do play a role in development of disease.

Taken together, it is important to explore new strategies in curbing the TB epidemic and this warrants further studies of previously underappreciated cell types in latent TB immunology, including B cells. We therefore focus on the non-humoral functional responses of B cells during antigenic challenge to shed light on B cell cytokine production, and the possible implications it might have on the host’s immune response to *M*.*tb*.

## Materials and Methods

### Ethics statement

Ethical approval for this study (including written informed consent) was obtained from the Health Research Ethics Committee of the University of Stellenbosch with reference #: N10/01/013 and N13/05/064. All the participants gave written informed consent to participate in the study.

### Study subjects

For this pilot study we recruited healthy male and female community controls (in year 2014) between the ages of 18 and 56, who were HIV negative and with no record of previous active tuberculosis disease. All participants in this pilot study were interferon gamma release assay (IGRA) (QuantiFERON (QFN) TB Gold) test positive (latent TB). Mantoux skin test was not used as a determinant of TB exposure as it is less specific than the QFN test. We created a TB contact score to evaluate each participant’s exposure to TB in the community. Low exposure was defined as living/working in an area with low TB burden, “medium exposure” was defined as living/working in an area with a known high TB burden and “high exposure” was scored when individuals lived or worked with someone with active TB disease. A total of eleven participants were recruited of which six (6/11; 54,5%) were male. Mean age and weight was 26±10.7 (years) and 61.1±11.3 (kilograms) respectively. Heparinized whole blood (70ml) was collected at recruitment.

### PBMC isolation, B cell enrichment and cryopreservation

Peripheral blood mononuclear cells (PBMCs) were isolated from whole blood by using Ficoll-Paque PLUS (GE Healthcare Life Sciences density gradient centrifugation. Cell counts were performed using the Trypan Blue cell exclusion method. B cell enrichment was done using the MACS beads technology from Miltenyi Biotec through negative-selection (human B cell isolation kit II). Enriched B cells were subsequently stored in cryo media (90% FCS and 10% DMSO) at -80°C. The purity of the enriched B cells from PBMCs, following the MACS beads protocol, were assessed utilising anti-human CD3 (PerCP, clone UCHT1, eBioscience and anti-human CD19 (FITC, clone HIB19, eBioscience) by FACS analysis, with resulting purities above 90%.

### B cell culture

Various antigens were utilised to stimulate the B cells to assess their respective responses. These antigens included: Phytohaemagglutinin (PHA, Sigma Aldrich) at 0.5mg/ml, purified protein derivative (PPD, Statens Serum Institut) at 12.5μg/ml, Lipopolysaccharides (LPS, Sigma) at 1mg/ml, Bacillus Calmette–Guérin (BCG ID Vaccine (*M*. *Bovis* BCG), Statens Serum Institut) at 6x10^6^ cfu/ml and the Toll-like receptor 9 agonist (TLR9a, Miltenyi Biotec) at 0.5μM. B cells were incubated at 37°C and 5% CO_2_ for 16 hours. Cells were cultured in complete media consisting of RPMI (Sigma) supplemented with 10% FCS and 2mM L-Glutamine (Sigma). B cells (5×10^5^cells/well) were cultured for each stimulation, in the presence of Brefeldin A (Sigma), for flow cytometry. Supernatants were collected from B cells, which were cultured in parallel without Brefeldin A, and stored at -80°C for multiplex analysis.

### Multiplex cytokine Analysis

The quantification of secreted molecules in the 16-hour culture supernatants including IFN-γ, IL-1β, IL-2, IL-4, IL-6, IL-8, IL-10, IL-12p70, IL-13 and TNF-α was determined using the Meso Scale Discovery (MSD^®^) platform. Experiments were performed strictly as recommended by the manufacturer after which plates were read on a Quickplex SQ 120 instrument (MSD).

### Flow cytometry

For intracellular staining, cells were cultured with Brefeldin A (Sigma) at a concentration of 10μg/ml for the duration of the stimulation. Cultured B cells were firstly stained with antibodies against cell surface markers (CD3, CD19, CD27 and CD138 –all from eBioscience) for 20 minutes, washed with FACS staining buffer (PBS, 2% FCS) and fixed and permeabilized using BD cytofix/cytoperm kit (BD Bioscience Pharmingen). These B cells were subsequently stained with antibodies against cytoplasmic proteins (IL-10, IL-17, IL-21 and TNF-α (eBioscience)) for 20 minutes in the dark and at room temperature, where after it was washed according to manufacturer’s instructions (BD Bioscience). A FACS Canto II (BD Bioscience) was used for cell acquisition (≥100,000 events). The instrument was calibrated according to the manufacturer’s instructions. Quality controls included the use of Rainbow Beads (eBioscience—San Diego, CA, USA) and the compensation settings were adjusted in conjunction with the use of antibody-capture beads (CompBeads, BD Biosciences) [25]. Fluorescence-minus-one (FMO) control samples were utilised (as described by Perfetto *et al*. [26]) to determine appropriate gating cut-off, to increase the accuracy of distinguishing different populations.

### Data and statistical analysis

Data analysis for the MSD results were done using Statistica 12, Statsoft (Ohio, USA). One-way ANOVA was done and the 95% confidence intervals plotted. Unbiased hierarchal clustering of cytokine secretion from the MSD experiments (across all stimulating conditions) and the generation of a heat map was done using Qlucore Omics Explorer (Lund, Sweden). QIAGEN’s Ingenuity Pathway Analysis software (Limburg, Netherlands) was utilised to assess the involvement of differentially-expressed cytokines in immunological pathways of diseases and disorders. Wilcoxon rank sum tests, where p-value adjustment was done using the Bonferroni post-hoc test, was used for the FACS dataset. All FACS plots were analysed in Flowjo and the data analysis were performed in R (http://www.r-project.org).

## Results

### LPS and TLR9-a stimulation results in the highest cytokine-secretion upregulation

**Fig 1:**
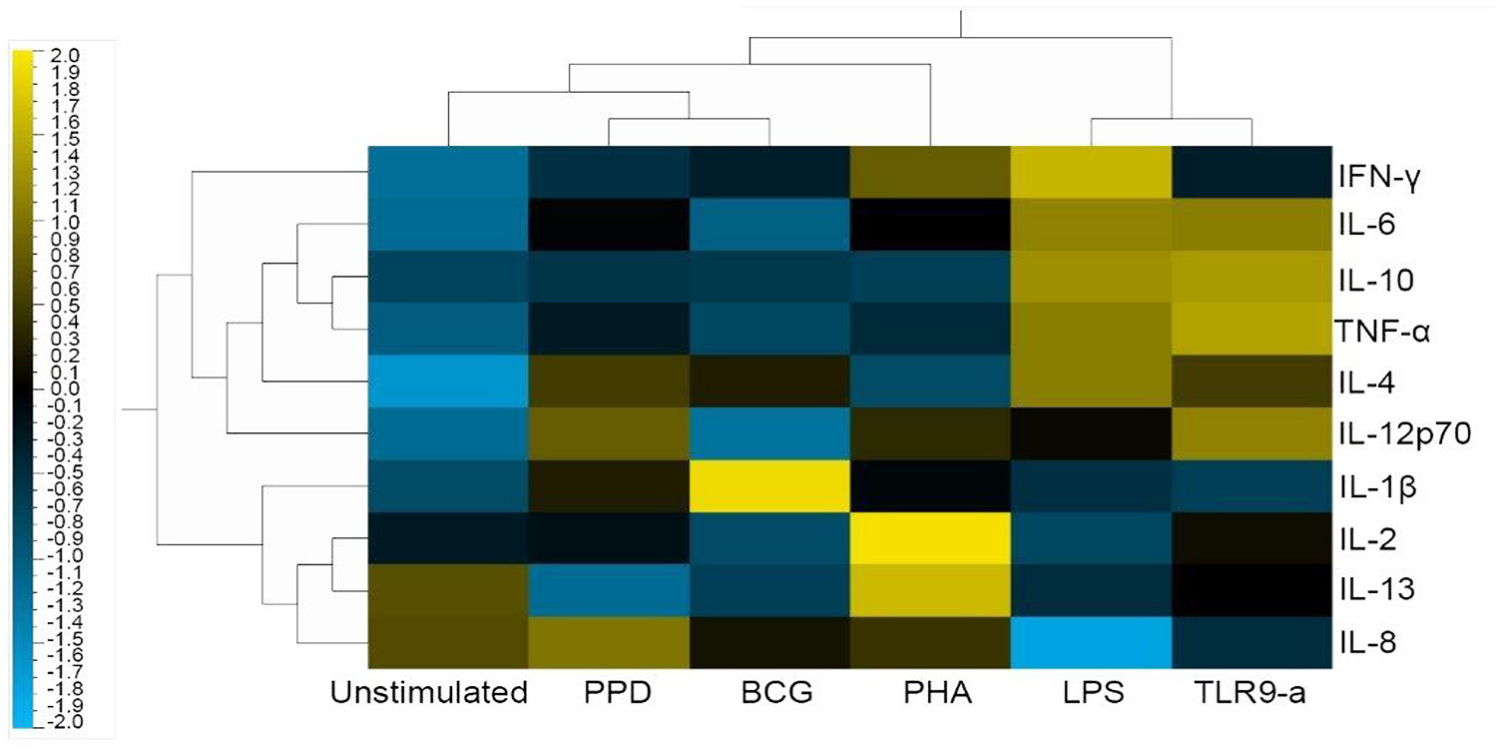
Differential secretion of cytokines in B cell supernatant following a 16-hour stimulation with multiple antigens. Qlucore Omics explorer software was used to do an unbiased hierarchical analysis and generate a heat map where cytokines were clustered based on expression within each stimulating condition. Blue represents cytokines under-expressed while yellow represents cytokines being over-expressed.

Given the importance of cytokine mediated responses during TB, we investigated the functional capacity of B cells from latently infected individuals by stimulating B cells with a broad range of antigens (from B cell-specific to TB-specific antigens) *in vitro*. The concentration of cytokines was assessed in the B cell culture supernatants after antigen stimulation using a MSD pro-inflammatory panel. In an unbiased analysis approach, Qlucore Omics Explorer software was used to generate a heat map of the antigen induced cytokine responses (Fig 1). From the heat map it is evident that LPS and TLR9-a were strong inducers of pro-inflammatory markers in B cells with markers such as IL-6, IL-10 and TNF-α being upregulated. LPS stimulation further resulted in the upregulation of IL-4 whereas TLR9-a stimulation resulted in the upregulation of IL-12p70. BCG induced strong IL-1β responses and PHA strong IL-2 and IL-13 responses in B cells. When comparing B cell responses to whole blood response (in QFN supernatants), an almost complete opposite was true. IL-10 and IL-6 were downregulated, in whole blood stimulated with the *M*.*tb* antigens ESAT-6/CFP-10/TB-7.7, TNF-α had no change in expression and IL-4 was upregulated but to a larger extent than LPS (data not shown). Furthermore, cytokines produced from these B cells are upregulated at levels which are comparable to T cell derived sources (data not shown).

### B cells produce differential pro-inflammatory cytokines profiles in an antigen-dependent manner

**Fig 2:**
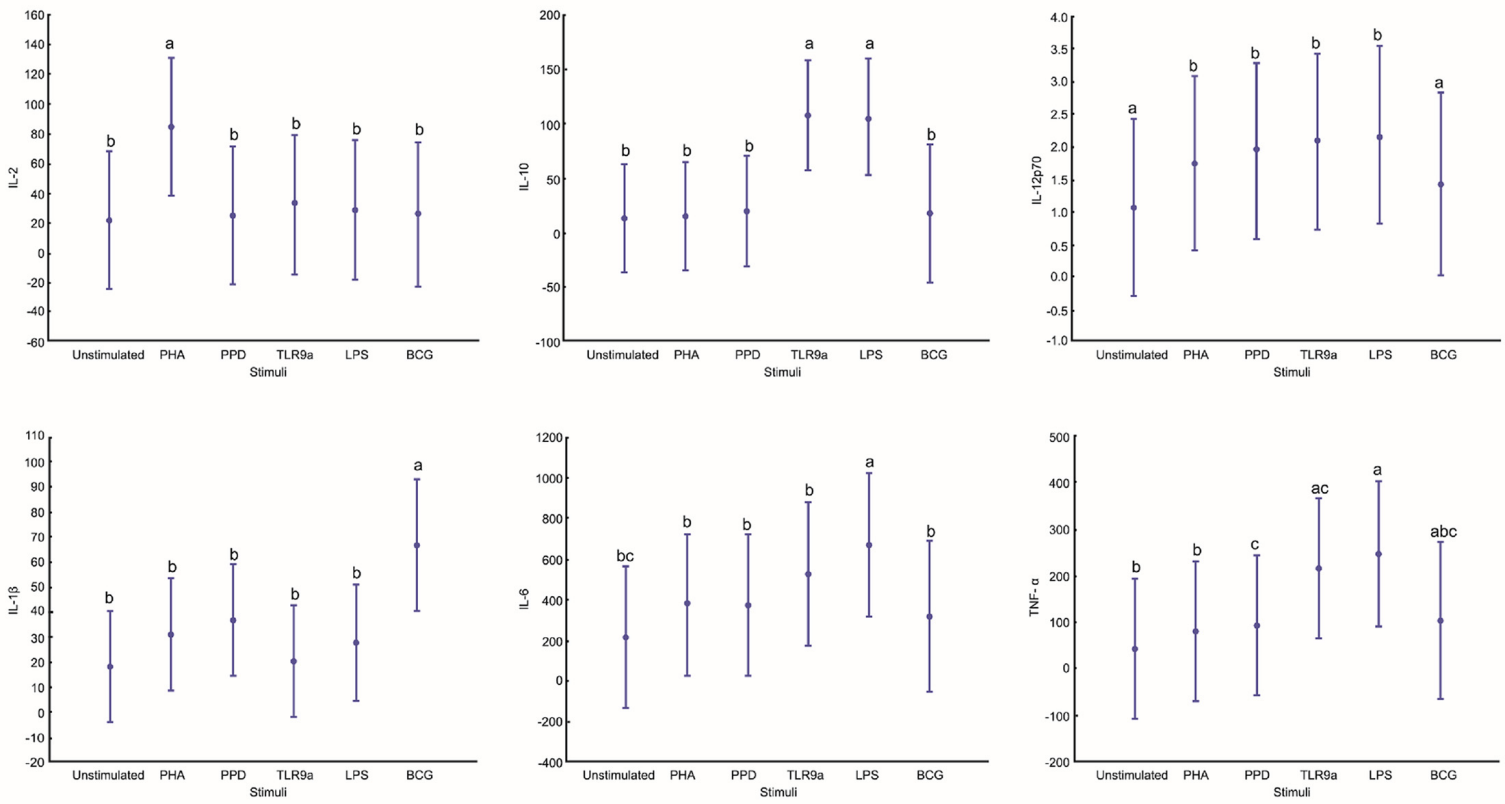
B cells produce pro-inflammatory cytokines differentially based on stimulation. B cells were stimulated for 16 hours in the presence of various antigen before the supernatant was analysed on the MSD platform to quantify specific pro-inflammatory cytokine. Points on the graph with commonly shared letters indicate no significant difference between those points. Y-axis represent cytokines levels in pg/ml. A confidence interval of 95% is depicted.

Univariate analysis showed that significant differences were found in six of the ten pro-inflammatory markers assessed (Fig 2). It is well documented that B cells produce IL-10, and here we show that LPS and TLR9-a stimulation induce significantly higher levels, with p ≤ 0.02 and p ≤ 0.01 respectively, when compared to the unstimulated and the other antigens. PPD (p = 0.024 versus LPS), TLR9-a (p ≤ 0.01 versus US) and LPS (p ≤ US, PHA and PPD) induced significantly higher concentrations of TNF-α (Fig 2). The highest TNF-α concentration was observed when B cells were stimulated with LPS. A similar trend was observed with LPS inducing significantly higher concentrations of IL-6. BCG induced significantly higher concentrations of IL-1β when compared to all other stimulants (p ≤ 0.001 versus US). BCG however did not induce IL-12p70 responses in these cells (p ≤ 0.01 for each one respectively). PHA induced significantly higher concentrations of IL-2.

### B cell IL-1β production primarily facilitates intra-cellular communication

**Fig 3:**
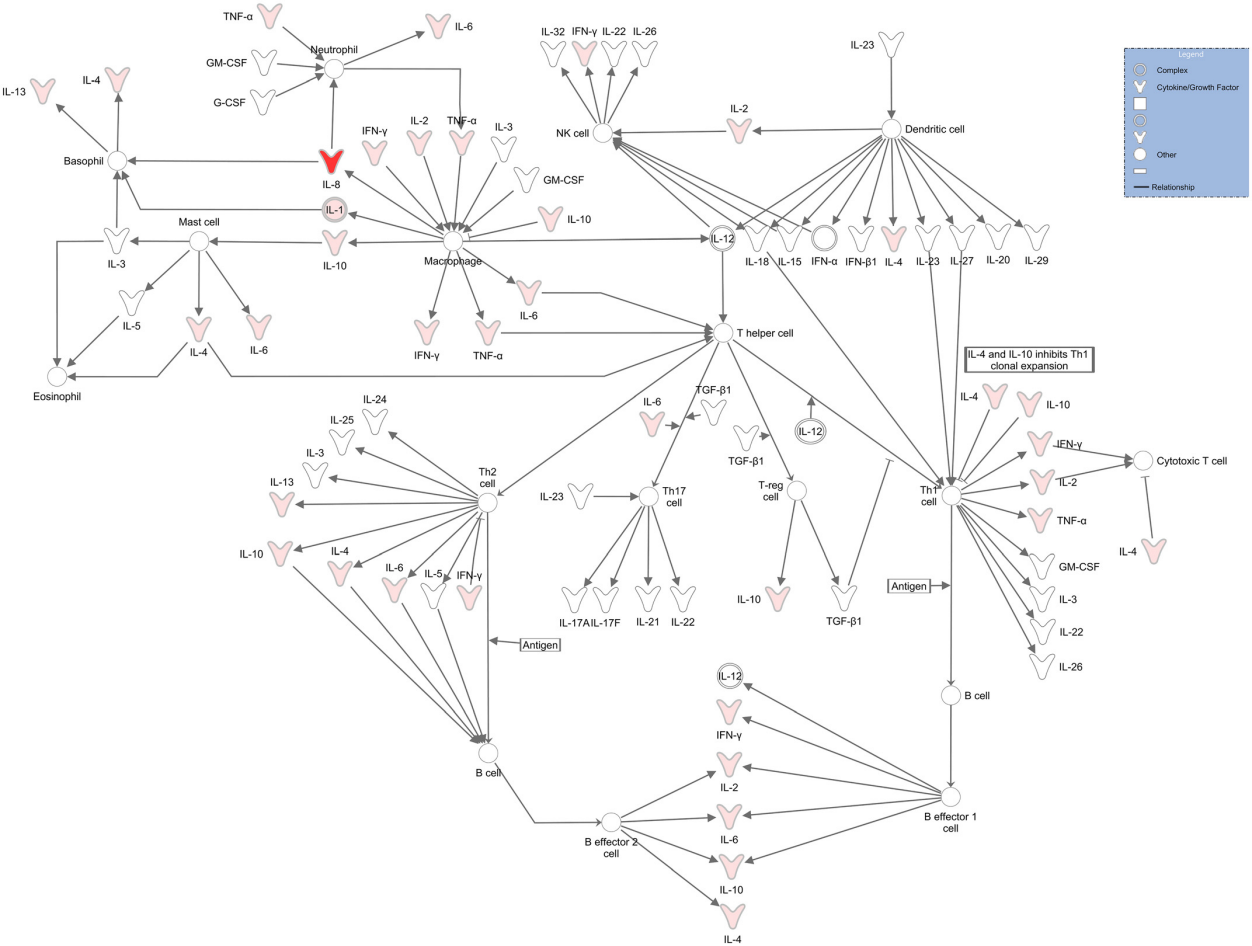
B cell cytokine production is primarily implicated in mediating communication between immune cells. Ingenuity pathway analysis (IPA) software indicated that the upregulation of IL-1β following BCG stimulation are primarily implicated in pathways which facilitate cellular communication between immune cells, with a p-value of 5,83E−11. The second top pathway it’s implicated in is T-helper cell differentiation with a p-value of 1,65E−10, and is also implicated in the communication between innate and adaptive immune cells with a p-value of 3,77E−10. Purple boxes represent markers that are from the dataset and participate in the pathway. Red boxes indicate markers present in the dataset that are upregulated in the pathway. White boxes indicate markers present in the pathway, but not in the dataset.

To follow on the over-expression of IL-1β during BCG stimulation, we analysed the respective dataset using the Ingenuity Pathway Analysis software (IPA, QIAGEN Redwood City, www.qiagen.com/ingenuity) and found that the top pathway associated was the role which cytokines play in mediating communication between immune cells (Fig 3). The only cytokine from our dataset which was broadly induced was IL-8 (Figs 1 and 3). What is interesting is that IL-4, IL-6 and IL-10 which are upregulated in our B cell dataset is implicated in the communication with B cells (Table 1 summarizes the top five pathways B cell derived IL-1β is implicated in), with further downstream effector functions from “B effector 2 cells”. These in turn produce more cytokines which are present in our dataset, suggesting a feed-forward mechanism. Our results suggest that B cell derived cytokines communicate and influence immune cells which play a key role in TB immunity. These cytokines are produced independent from the traditional T cell based sources, and could function as pro-inflammatory or anti-inflammatory effectors with possible feed-forward mechanisms, which boosts the effect.

**Table 1:** Top five pathways B cell derived IL-1β is implicated in.

Stimulant	Marker	Top 5 Pathways	p—Value
BCG	IL-1β	1. Role of Cytokines in Mediating Communication between Immune Cells	5,83E-11
		2. T Helper Cell Differentiation	1,65E-10
		3. Altered T Cell and B Cell Signalling in Rheumatoid Arthritis	3,58E-10
		4. Communication between Innate and Adaptive Immune Cells	3,77E-10
		5. Role of Pattern Recognition Receptors in Recognition of Bacteria and Viruses	1,71E-09

### Plasma (memory) B cells are the primary source of B cell-derived cytokines

**Fig 4:**
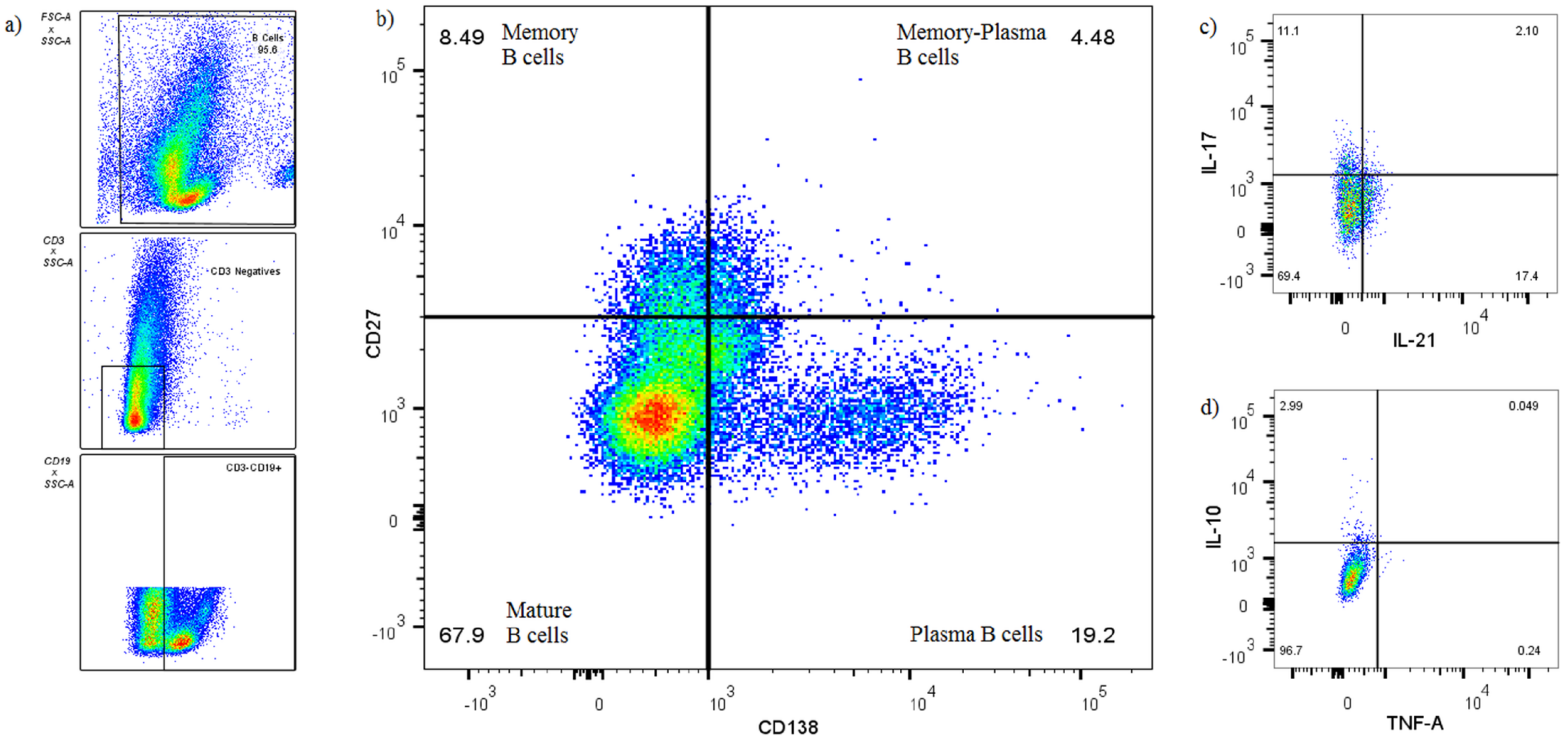
B cell flow cytometry gating strategy. a) Initial cell- inclusion and exclusion gating. b) The main B cell phenotypes from which cytokine production were assessed includes memory B cells (CD19^+^CD27^+^), mature B cells (CD19^+^CD27^-^CD138^-^), plasma B cells (CD19^+^CD138^+^) and plasma (memory) B cells (CD19^+^CD27^+^CD138^+^). c) Further downstream cytokine analysis were done following the identification of the main B cell subsets in b), by plotting respective cytokines against one another. Appropriate cut-off levels for the gating strategy were used as determined by using FMO control samples.

**Fig 5:**
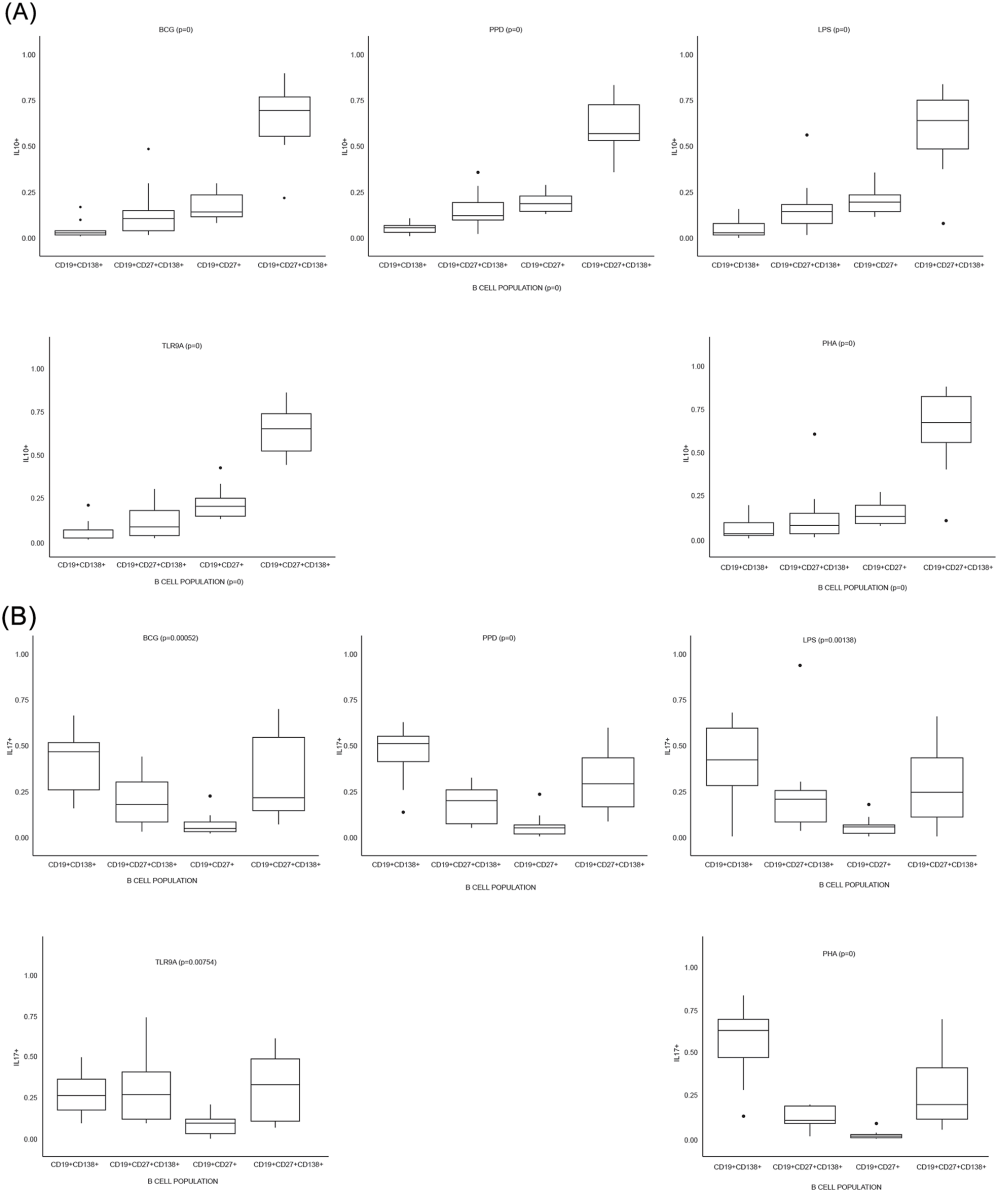
Plasma (memory) B cells (CD19^+^CD27^+^CD138^+^) are major contributors to cytokine production. B cells were stimulated for 16-hours with various antigens, whereafter flow cytometric analysis were performed to assess intracellular cytokine production of specific B cell subsets. Each box-plot represents the relative cytokine production as contributed by the respective B cell subset, and according to stimulating condition. The contribution of each B cell subset in a graph adds up to 100% of the total respective cytokine’s production, for that stimulating condition. Graphs are included for the frequency of the four cytokine measured, including (A) IL-10 and (B) IL-17, (C) IL-21 and (D) TNF-α.

**Fig 6:**
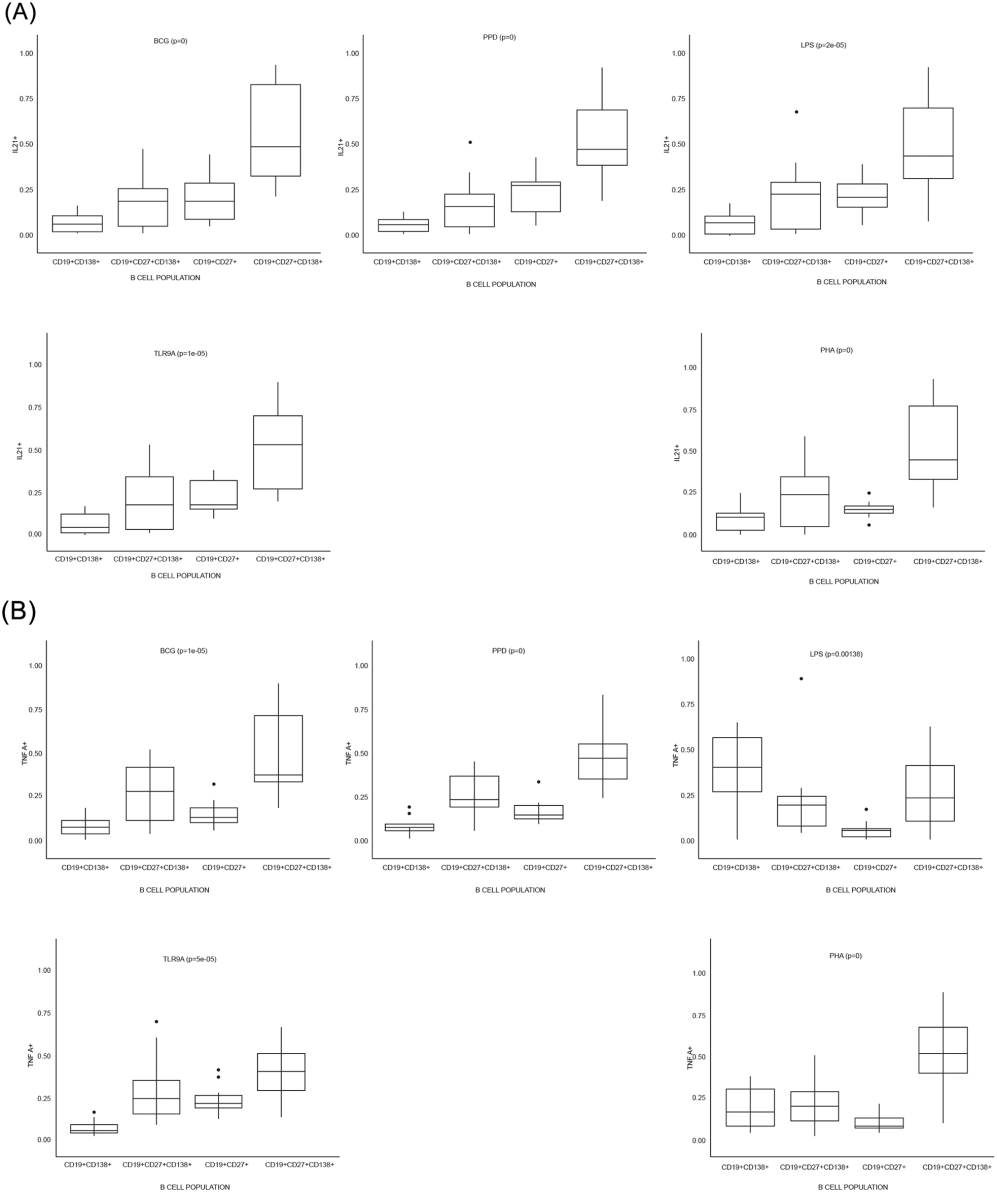
Plasma (memory) B cells (CD19^+^CD27^+^CD138^+^) are major contributors to cytokine production. B cells were stimulated for 16-hours with various antigens, whereafter flow cytometric analysis were performed to assess intracellular cytokine production of specific B cell subsets. Each box-plot represents the relative cytokine production as contributed by the respective B cell subset, and according to stimulating condition. The contribution of each B cell subset in a graph adds up to 100% of the total respective cytokine’s production, for that stimulating condition. Graphs are included for the frequency of the four cytokine measured, including (A) IL-21 and (B) TNF-α.

**Fig 7:**
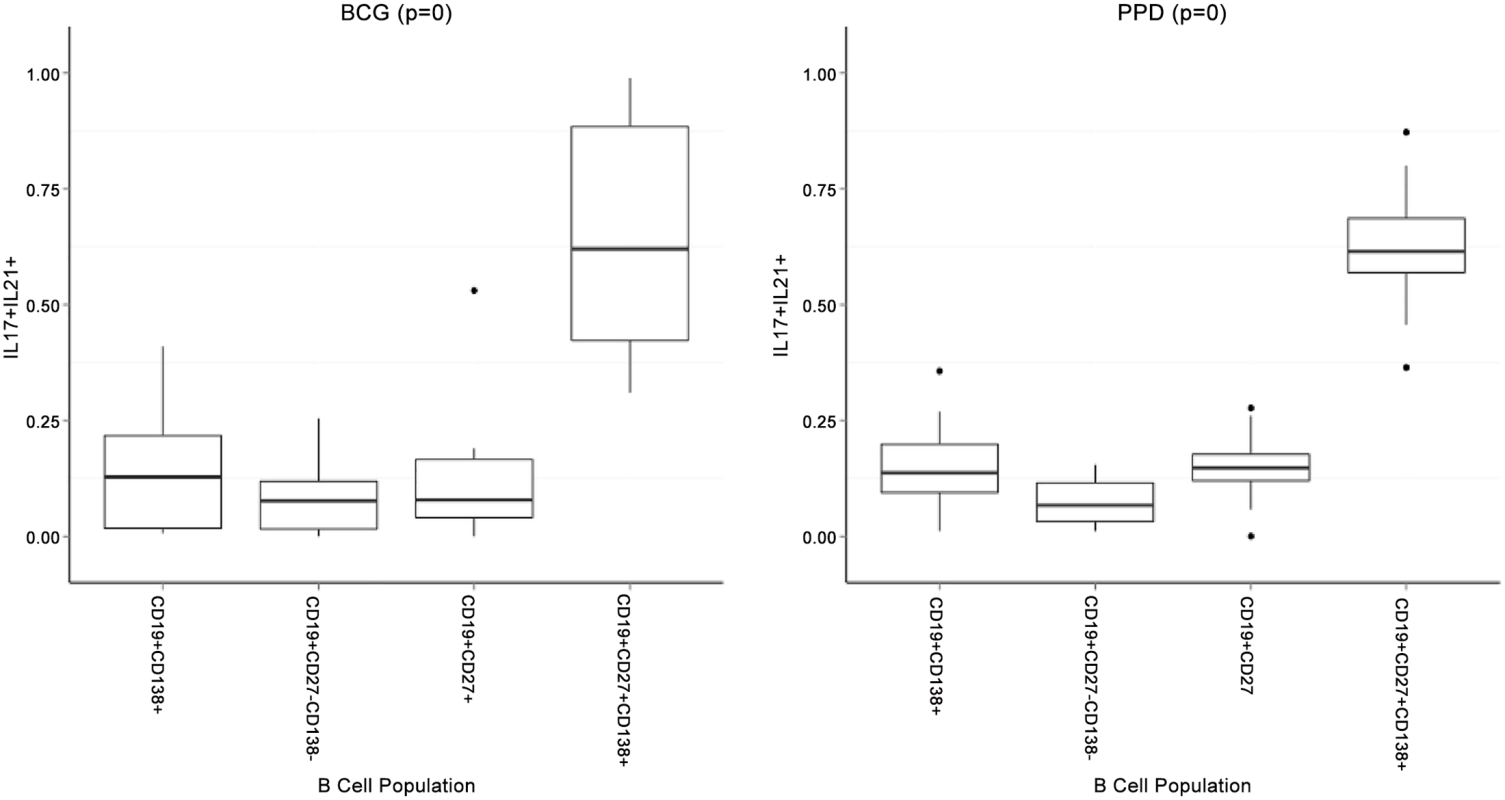
Plasma (memory) B cells may be key B cells subsets during innate recruitment of B cells during tuberculous challenge. B cells were stimulated for 16-hours with BCG and PPD respectively. Flow cytometric analysis of various B cell subsets indicated that plasma (memory) B cells were the major contributors for the dual production of IL-17 and IL-21, implicating them in the potential recruitment of innate-like B cells during *M*.*tb* challenge. Each box-plot represents a fraction from each B cell subset to the total cytokine production.

After assessing the general functional capacity of B cells, we wanted to assess in more depth which specific B cell subsets contributed the most to cytokine production following antigenic challenge. By using FACS four B cell populations were identified based on their cytokine production profile. These were defined as: plasma (PB) B cells (CD19^+^CD138^+^), mature B cells (CD19^+^CD27^-^CD138^-^), memory (MB) B cells (CD19^+^CD27^+^) and plasma (memory) (M-P) B cells (CD19^+^CD27^+^CD138^+^). Fig 4 shows the gating strategy followed to acquire these B cell populations, and the cytokine production profiles. The graphs represented in Figs 5 and 6 respectively indicates total cytokine production as contributed by each subset, where the total combined contribution adds up to 100% of production. Four cytokines, namely IL-10, IL-17, IL-21 and TNF-α, and combinations thereof were measured (Figs 5 and 6). From our results it is clear that plasma (memory) B cells (CD19^+^CD27^+^CD138^+^) are major contributors of cytokines compared to other subsets. This is especially true for IL-10 production, where plasma (memory) B cells produced significantly more (p ≤ 0.001, and in some cases p ≤ 0.0001) cytokines than any other subset. There seems to be a trend to cytokine production within the subsets, with the least cytokine being produced by plasma B cells (CD19^+^CD138^+^), followed by mature B cells (CD19^+^CD27^-^CD138^-^) and memory B cells (CD19^+^CD27^+^) producing the second most. Interestingly, the inverse is observed when considering IL-17 production with plasma B cells being the top contributor (p ≤ 0.05 when compared to other B cell subsets within the majority of stimulations) followed by plasma (memory) cells in second place. PPD (p = 0.0023, compared to mature B cells) and BCG (p = 0.0003, compared to memory B cells) induced significantly more IL-17 than TLR9-a, which was the greatest contributing stimulant of secreted cytokine production (Figs 1 and 2). The plasma (memory) B cell subset also had the highest frequency of IL-17^+^IL-21^+^ cells, with p ≤ 0.001 compared to all other populations, for both BCG and PPD (Fig 7).

## Discussion

It is estimated that about 2 billion individuals worldwide are infected with latent tuberculosis [27]. Although only 1 out of every 10 individuals will progress to active disease in their lifetime, people latently infected with tuberculosis still serve the purpose of acting as seedbeds for future cases of active tuberculosis disease. There has been an increase in the literature describing new functional capacities of B cells and, although great progress has been made in delineating these functions, the full functional repertoire of these cells still remains incompletely described. Here we aimed to assess the functional response of B cells to various stimuli within the milieu of latent tuberculosis infection to determine a) if they responded in a non-humoral capacity to stimulation, b) to what extent they responded and, c) if B cells respond uniquely to TB-related stimulations. Understanding the non-humoral role of B cells during exposure to *M*.*tb* and the subsequent role they play in polarizing the T-cells is an important step in our understanding of the immune response to infectious disease.

B cells displayed a significant ability to produce IL-2, IL-6, IL-10, IL-12p70, IL-1β and TNF-α following antigenic stimulations as measured from culture supernatant, with a specific preference to stimulations originating from toll-like receptors (LPS and TLR9-a) with the exception of IL-1β that was only produced in significant quantities following whole organism (BCG) stimulation.

Toll like receptors (TLRs) contain germline-encoded receptors or pattern-recognition receptors (PRR), that are pivotal during the innate recognition of pathogen associated molecular patterns (PAMPs) [28]. B cells readily express a host of TLRs, including TLR4 and TLR9 [29], and although it is believed that *M*.*tb* is recognised by TLR1, TLR2, TLR4, TLR6, TLR8 and TLR9 (which recognises specific unmethylated CpG motifs prevalent in microbial DNA) [30], the majority of *M*.*tb* related TLR research has focussed of TLR2 and TLR4. Several groups have shown that polymorphisms in TLRs are associated with increased susceptibility to tuberculosis [30–33]. TLRs are not only implicated in TB disease susceptibility, but also crucial for host protection as a study on TLR4^-/-^ mice resulted in exacerbated and disseminated disease coupled with neutrophilia, reduced macrophage recruitment and poor outcome [34]. In contrast to a new study that shows a minor contribution from TLR9 and TLR2 in inducing memory immunity to *M*.*tb* with live vaccines [35], the findings of our study highlights the significance of TLR responses not only for the possible influence they might have within TB disease, but the possible link they fulfil between innate and adaptive responses by activating B cells on an innate level which results in intrinsic adaptive responses from the B cells with subsequent cytokine production.

While TLRs and C-type lectin receptors are responsible for recognising extracellular mycobacterial components, aryl hydrocarbon receptors (AhRs) and nucleotide-binding oligomerization domain-like receptors (NLRs) sense, and readily respond to mycobacterial molecules found intracellularly in the cytosol [36, 37]. The resulting signalling cascades from these receptors facilitate the production of pro-inflammatory molecules such as cytokines, chemokines and anti-microbial molecules that controls cell death [38]. These include cytokines which have proven to be critical in the host response against tuberculosis, including IL-12, TNF-α and IL-1β [39]. IL-1β is intricately connected to the inflammasome, which is commonly found in innate cells and functions as a molecular platform from which pro-inflammatory cytokines (such as IL-1β) can mature following activation caused by cellular- stresses and infection [40]. Inflammasomes are common amongst innate cells such as dendritic cells and macrophages [41], but whether they can be activated and function from within lymphocytes (such as B cells) remains unclear. Inflammasomes are, however, implicated in the host immune response against *M*.*tb* [42–45] and has recently been the target of a promising new TB vaccine that utilises recombinant BCG [46]. Our data shows that B cell stimulation with BCG, and not PPD, resulted in the significant production of IL-1β. This finding raises the question as to the importance of whole-bug presence in the successful activation of the inflammasome platform during mycobacterial challenge.

Although a lot of work has been done in identifying both the pro- and anti-inflammatory capacity of B cells [20, 47–50], the knowledge regarding which specific phenotypic subsets of B cells are responsible for the production of specific cytokines is incompletely described. Here we aimed to not only demonstrate B cell cytokine production following specific stimulation, but to shed light on the phenotype of the major cytokine producing subsets. Several studies have focussed on the regulatory functions of B cells (those producing IL-10 and IL-35) and subsequently showed that antibody secreting B cells with a CD19^+^CD138^hi^ phenotype were the primary producers of IL-10 and IL-35 [47, 51, 52]. Here we show for the first time that IL-10 is produced in significantly greater quantities by plasma (memory) B cells (CD19^+^CD27^+^CD138^+^) when compared to plasma B cells (CD19^+^CD138^+^), regardless of stimulating condition. This finding places new emphasis on accurately distinguishing between specific B cell subsets when assessing function and possible downstream effects. We further demonstrated that plasma (memory) B cells are also the drivers of TNF-α and IL-21 production. TNF-α is produced by many cell types and has shown to have good cytotoxic synergy with human interferon [53], where TNF-α is required for protective immunity in mice [54]. Plasmablasts are inherently short lived, but the effects of TNF-α on these cells suggests that their lifetime can be extended [55]. TNF-α is also required for the formation and maintenance of granulomas and can even influence the production of chemokines during *M*.*tb* challenge [56–59]. IL-21 is a cytokine with pleiotropic effects, which includes the differentiation of naïve- and memory B cells into plasma cells, as well as having the capacity to induce the maturity of CD8^+^ T cells with enhanced cytotoxicity [60]. IL-21R was shown to be expressed on germinal centre and naïve B cells, but not on memory- or plasma B cells [61]. Interestingly, we found that the frequency of B cell derived IL-21 was the greatest when compared to the other cytokines in our dataset (data not shown). This would suggest that B cells, and specifically memory- or plasma B cells, regulate the activation and maturation of more B cells *via* an autocrine loop following antigenic stimulation. These cytokine also has huge implications also for the maintenance of *M*.*tb*-specific CD8 T cells in humans. Another interesting finding was that IL-17 was predominantly produced by plasma B cells (CD19^+^CD138^+^), and not plasma (memory) B cells like the other cytokines and these findings are in accordance with Bermejo *et al*. [62]. The observed IL-17 production from B cells could be activated independently from the usual major requirements for IL-17 production. This included IL-6, IL-23, AhR, RoRgt and RORa for T cells; and independent of MyD88 and CD40, who are both major pathways of activation in B cells [62]. Our findings reinforce the notion that B cells could function in the innate control of *M*.*tb via* IL-17 production.

Our pilot study suggests that B cells readily respond to a host of stimulations in a non-humoral manner. These findings warrant further research into the functional capacity of B cells within the tuberculosis milieu, with a focus on specific phenotypes and cell subsets. As this a pilot study in our community of predominantly latent TB infected individuals (about 2% TST negative in a study of 1400 participants) we first aimed to get an idea of how the B cells respond in our setting in “healthy” individuals. This study will have to be expanded to include QFN negative people and compare that to individuals with active TB disease. These larger studies are planned. Further characterization of these B cells using fresh samples must be carried out and compared to our study which was performed on frozen samples. Additionally, larger studies should also collect more samples (serum or supernatants) to test for the production of antibodies. The identification of B cell subsets or features that functionally produce pro-inflammatory and/or anti-inflammatory cytokines could pave the way for new strategies regarding vaccine development and B cell directed treatment in the clinic. For example, the selective depletion of B cell subsets implicated in disease pathology without affecting the remaining B cell subsets producing cytokines associated with TB disease resolution would be of great benefit to the patient.
